# Protein-protein interactions between lens vimentin and αB-crystallin using FRET acceptor photobleaching

**Published:** 2008-07-10

**Authors:** Shuhua Song, Mark J. Hanson, Bing-Fen Liu, Leo T. Chylack, Jack J-N. Liang

**Affiliations:** Center for Ophthalmic Research/Surgery, Brigham and Women's Hospital, and Department of Ophthalmology, Harvard Medical School, Boston, MA

## Abstract

**Purpose:**

The R120G mutation of αB-crystallin is known to cause desmin-related myopathy, but the mechanisms underlying the formation of cataract are not clearly established. We hypothesize that alteration of protein–protein interaction between R120G αB-crystallin and lens intermediate filament proteins is one of the mechanisms of congenital cataract.

**Methods:**

Protein–protein interactions were determined by confocal fluorescence resonance energy transfer (FRET) microscopy using green fluorescence protein (GFP) as the donor and red fluorescence protein (RFP) as the acceptor. The lens vimentin gene was fused into a GFP vector and the αB-crystallin (WT or R120G mutant) gene was fused into the RFP vector. The donor-acceptor plasmid pairs of intermediate filament (IF)-GFP and αB-RFP were co-transfected into HeLa cells. After incubation, confocal fluorescence images of the transfected cells were taken. FRET was estimated by the acceptor photobleaching method. Protein–protein interaction was evaluated by FRET efficiency.

**Results:**

The confocal fluorescence images showed that the cells expressing vimentin and R120G αB-crystallin contained large amounts of protein aggregates while few vimentin fibers were observed. FRET efficiency analyses indicated that vimentin had a significantly greater protein–protein interaction with R120G αB-crystallin than with WT αB-crystallin.

**Conclusions:**

Our results show that the R120G αB-crystallin mutant promoted vimentin aggregation through increased protein–protein interaction. This process may contribute to the formation of congenital cataract.

## Introduction

The lens cytoskeleton is composed of microfilaments, intermediate filaments (IFs), and microtubules [1-3]. The major function of IFs is to support cellular membranes and to serve a structural role in maintaining cell shape. The lens fiber cell contains three IFs: vimentin, CP49, and filensin [1,4-6]. The latter two are lens-specific and form beaded filaments. Vimentin is a type III intermediate filament. Among the various crystallins, αB-crystallin is most closely associated with IF proteins, not only in lens fiber cells [7-9] but also in muscle cells [10,11]. Desmin, also a type-III IF protein found mainly in smooth and cardiac muscle cells [12], has been the subject of extensive study in desmin-related myopathy (DRM), an adult-onset neuromuscular disease characterized by large accumulations of aggregates of cytoplasmic desmin and R120G mutant αB-crystallin [13]. Patients with DRM show muscular weakness and present with cataracts [14-16]. Studies have demonstrated that muscle cell lines transfected with the mutant αB-crystallin cDNA show intracellular aggregates that contain both desmin and the αB-crystallin mutant [10,17,18]. Other studies have shown that the R120G mutation alters the interaction between αB-crystallin and IFs that may have contributed to DRM [11,19,20].

Although the link between desmin and the R120G αB-crystallin mutation is strong in DRM, it is less clear that the induction of cataract by this mutation is due to the same mechanism of association of R120G αB-crystallin and lens IFs. Moreover, lens fiber cells have vimentin, CP49, and filensin but not desmin. Vimentin is present in epithelial and cortical fibers, but it is absent in nuclear fiber cells [21]. Many studies have demonstrated an association between vimentin and αB-crystallin [7-9]. The other two IF proteins, filensin and CP49, assemble as a beaded filament, and their proper assembly also requires αB-crystallin [4,7]. In the present study, we have investigated the effects of the αB-crystallin R120G mutation on the protein–protein interaction with vimentin using confocal fluorescence resonance energy transfer (FRET) microscopy. FRET acceptor photobleaching was applied, and FRET efficiency values were obtained. Our results show that the αB-crystallin R120G mutant promotes aggregation of vimentin by increased protein–protein interactions.

## Methods

### Preparation of GFP and RFP fusion proteins

As in our previous studies, Clontech’s (Palo Alto, CA) pAcGFP-C1 and pDsRED Monomer-C1 vectors were used [22,23]. The pAcGFP1-C1 vector is encoded with a green fluorescent protein (GFP) gene from *Aequorea coerulescens* (λ_ex_/λ_em_=475/505 nm). The pDsRED-Monomer-C1 is encoded with a DsRED-Monomer gene with a red fluorescence protein (RFP), DsRED, a mutant derived from tetrameric *Discosoma* (λ_ex_/λ_em_=557/585 nm). The vimentin gene (in pBluescript vector) was obtained from ATTC (Manassas, VA). It was subcloned into the pAcGFP1-C1 vector by polymerase chain reaction (PCR) using the forward primer-CCT AAG CTT TGT CCA CCA GGT CC-containing the HindIII restriction site (underlined) and the reverse primer-CCC GAA TTC TTA TTC AAG GTC ATC-containing the EcoRI restriction site (underlined). The resulting construct was designated as GFP-VIM; its sequence was verified. The wild-type (WT) and R120G αB-crystallin constructs (RFP-αB and RFP-αBm [where m is R120G mutation]) were previously prepared [22].

### Transfection and cell culture

HeLa cells were cultured using the protocol described in our recent report [22]. Briefly, HeLa cells were seeded into a 35 mm culture dish. After culturing for 24 h to obtain at least 80% confluence, cells were co-transfected with the two constructs using the lipofectamine 2000 reagent (Invitrogen, Rockville, MD) at a ratio of cDNA:lipofectamine being 1:2. For a positive control, GFP-αA- and RFP-αB-crystallin were used, and for a negative control, GFP and RFP were used. After incubation for 48 h, cell images in the green and red channels were acquired using a Zeiss Laser Scanning Microscope (LSM; 510 META Axioplan 2, Carl Zeiss Inc., Thornwood, NY) at the Harvard NeuroDiscovery Center (Harvard Medical School, Boston, MA).

### FRET acceptor photobleaching

This method measures energy transfer efficiency (E) and is directly related to the distance (*r*) separating a given donor and acceptor pair by the Föster Equation [24-26]:

E=1/[1+(*r*/R_0_)^6^] (Equation 1)

where R_0_ is the Föster distance at which the transfer efficiency is 50%. The efficiency of transfer (E) can be calculated from the equation:

E=1–F_DA_/F_D_ (Equation 2)

where F_DA_ and F_D_ are the donor fluorescence intensities in the presence and absence, respectively, of energy transfer.

FRET acceptor photobleaching (FRET-AP) involves measuring the donor “de-quenching” in the presence of an acceptor. This is done by comparing the donor fluorescence intensity in the same sample (either a whole cell or region of interest [ROI] of a cell) before and after destroying the acceptor by photobleaching. If FRET was initially present, a resultant increase in donor fluorescence occurs upon photobleaching of the acceptor. The energy transfer efficiency is quantified by rewriting Equation 2 as:

E=1–F_pre_/F_post_ (Equation 3)

where F_pre_ is the fluorescence intensity of the donor before the acceptor photobleaching, and F_post_ is the fluorescence intensity of the donor after the acceptor photobleaching.

In the photobleaching experiments, a repetitive bleaching (at excitation wavelength of 543 nm) was applied to bleach the RFP signal in a ROI or a whole cell. A series of pre-bleaching and post-bleaching donor GFP fluorescence intensities were collected. The maximum and minimum values (GFP-max and GFP-min) were used for calculation of FRET efficiency by rewriting Equation 3 as:

E=1 – F_GFP-min_/F_GFP-max_ (Equation 4)

### Statistical analyses

Data are expressed as the mean±SEM from a minimum of three independent experiments. Statistical analysis was performed with either a Student’s *t*-test (two groups) or an ANOVA analysis (more than two groups) with p<0.05 as the criterion of significance.

## Results

**Figure 1:**
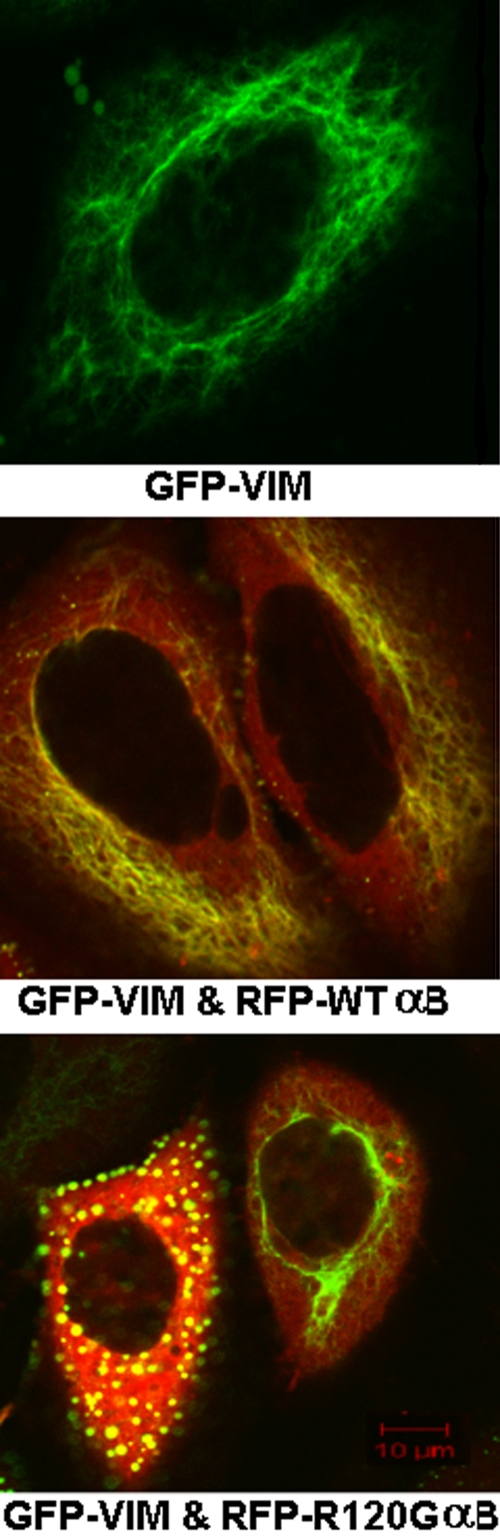
Representative laser scanning microscopy images of HeLa cells transfected with GFP-VIM and co-transfected with GFP-VIM and WT αB-crystallin (αB) or R120G αB-crystallin (αBM). The single construct or pair of constructs was transfected into HeLa cells. After culture, laser scanning microscopy (LSM) images were taken. Either the green image (GFP-VIM) or merged image of green and red fluorescence (GFP-VIM and RFP-WT αB-crystallin [αB] or GFP-VIM and RFP-R120G αB) was shown. Vimentin filaments are clearly shown in cells co-expressing WT αB-crystallin, but enormous aggregates were formed in the cells co-expressing R120G αB-crystallin. Vimentin filaments are shown as the fibrous structures and aggregates as the bright, dense spots.

Figure 1 shows representative confocal images of cells transfected with GFP-VIM or co-transfected with either GFP-VIM and RFP-WTαB or GFP-VIM and RFP-R120GαB. In the cells expressing GFP-VIM and RFP-R120GαB, a dramatic increase in the number of aggregates was observed. In addition, fewer vimentin fibers were present. Aggregation is shown as bright, dense spots. Cells expressing vimentin alone show rare aggregates.

**Figure 2:**
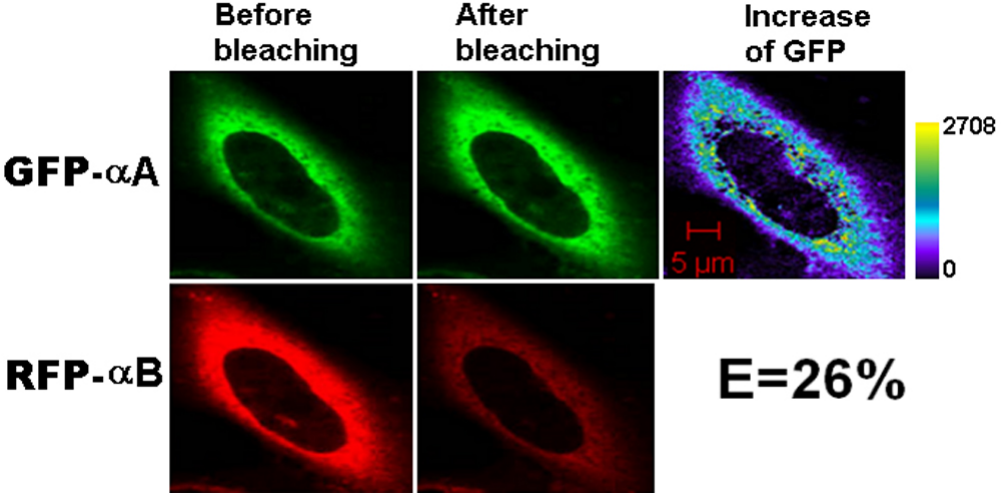
Representative laser scanning microscopy images of HeLa cells co-transfected with the positive controls, GFP-αA and RFP-αB. The constructs were co-transfected into HeLa cells. After culture, laser scanning microscopy (LSM) images were taken. αA- and αB-crystallins are known to have a strong subunit-subunit interaction. The energy transfer efficiency is high. The increase of GFP fluorescence intensity is converted to pseudocolor (right panel) that displays variations of pixel gray scales with color.

The fusion proteins, GFP-αA and RFP-αB, were used as a positive control (Figure 2) since αA-crystallin and αB-crystallin are known to have strong subunit-subunit interaction. We have reported a comparable FRET efficiency in a solution study [27]. In the photobleaching experiment, the acceptor is bleached, and as a result, acceptor fluorescence intensity shows a decrease and donor fluorescence intensity shows an increase since fewer acceptor chromophores are available for energy transfer. The pseudo-color images represent the increase of pixel density before and after bleaching. The color in the bar represents the pixel density of the image and thus the intensity of interaction.

**Figure 3:**
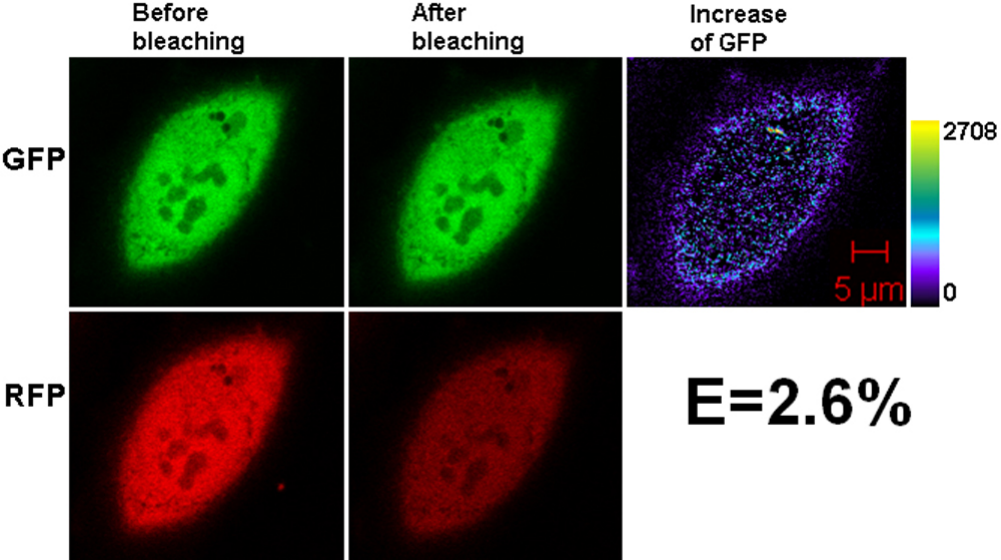
Representative laser scanning microscopy images of HeLa cells co-transfected with the negative controls, untagged GFP and RFP. The pair of constructs was co-transfected into HeLa cells. After culture, laser scanning microscopy (LSM) images were taken. The low efficiency shown arises from experimental background. The increase of GFP fluorescence intensity is converted to pseudocolor (right panel) that displays variations of pixel gray scales with color.

Nonfusion GFP and RFP were used as a negative control; they are not expected to interact. Figure 3 shows some representative confocal images. Theoretically, the negative control should show no increase of donor intensity after bleaching and thus no transfer efficiency, but in the acceptor photobleaching experiments, the donor is also affected. When repetitive bleaching is performed, donor intensity increases initially but then decreases slightly if there is energy transfer between the donor and acceptor. If there is no energy transfer, donor intensity shows a slight decrease, and some residual pixel density in Figure 3 is considered to be experimental background.

**Figure 4:**
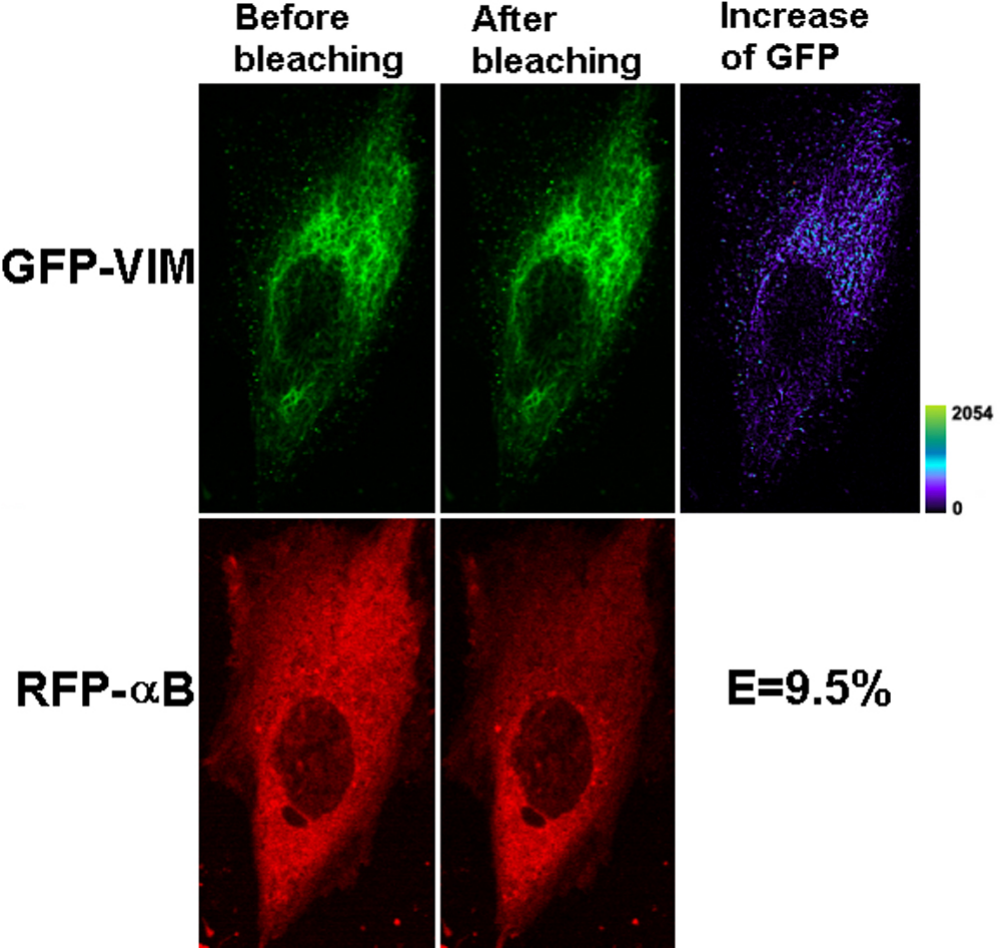
Representative laser scanning microscopy images of FRET acceptor photobleaching of HeLa cells co-transfected with GFP-VIM and RFP-WTαB. The pair of constructs was co-transfected into HeLa cells. After culture, laser scanning microscopy (LSM) images were taken before and after photobleaching of the acceptor for 45 s with a 543 nm laser beam. A decrease of red fluorescence and increase of green fluorescence were observed. The transfer efficiency was calculated with the equation: E=1 – F_GFP-min_/F_GFP-max._. The efficiency for this cell that co-transfected with GFP-VIM and RFP-WTαB is 9.5%, much greater than the negative control of untagged GFP and RFP. The increase of GFP fluorescence intensity is converted to pseudocolor (right panel) that displays variations of pixel gray scales with color.

**Figure 5:**
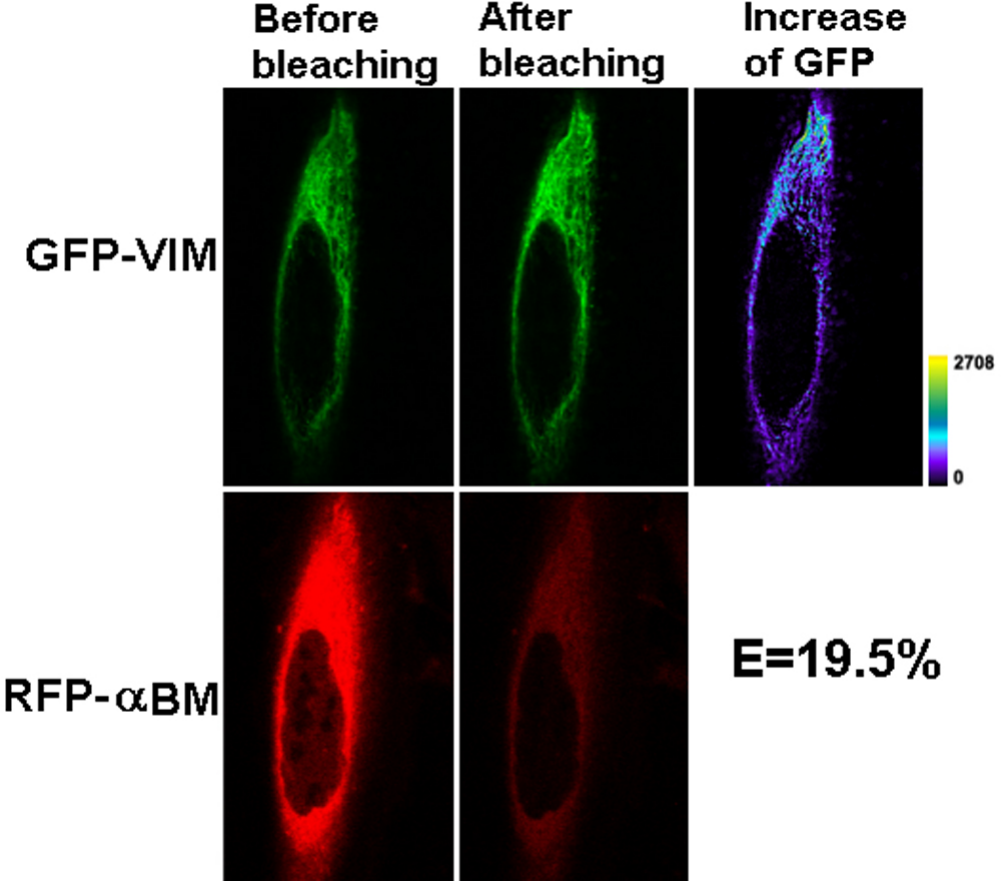
Representative laser scanning microscopy images of FRET acceptor photobleaching of HeLa cells co-transfected with GFP-VIM and RFP-R120G αB. The pair of constructs was co-transfected into HeLa cells. After culture, laser scanning microscopy (LSM) images were taken before and after photobleaching of the acceptor for 45 s with a 543 nm laser beam. A decrease of red fluorescence and increase of green fluorescence were observed. The transfer efficiency was calculated with the equation: E=1 – F_GFP-min_/F_GFP-max_. The efficiency for this cell that co-transfected with GFP-VIM and RFP-R120G αB is 19.5%, twofold greater than the GFP-VIM and RFP-WTαB. The increase of GFP fluorescence intensity is converted to pseudocolor (right panel) that displays variations of pixel gray scales with color.

**Figure 6:**
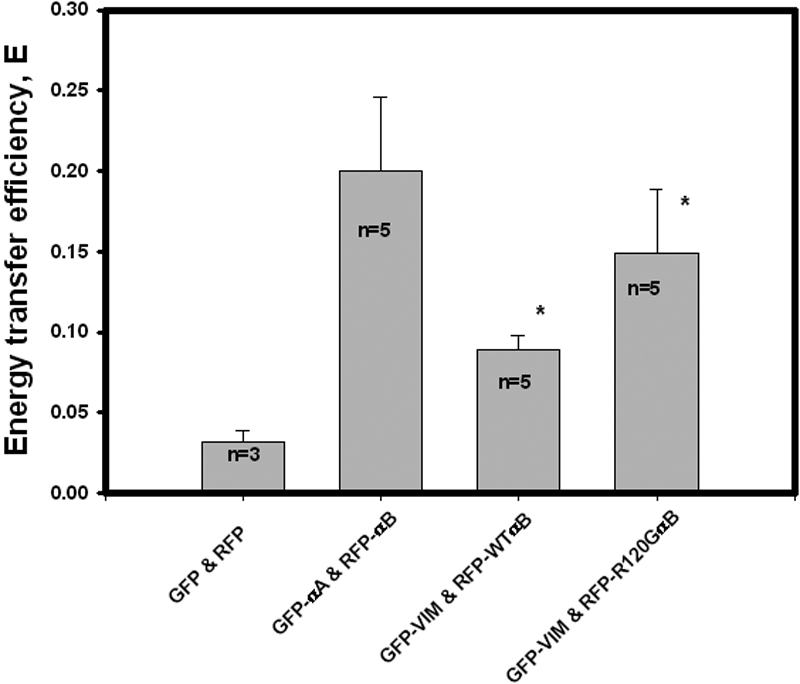
Summary of transfer efficiencies for various pairs, GFP and RFP, GFP-αA and RFP-αB, GFP-VIM, and RFP-WTαB and GFP-VIM and RFP-R120G αB. Significant differences in transfer efficiency were observed among them (ANOVA test, p=0.0013), and a twofold increase for the pair of GFP-VIM and RFP-R120G αB over the pair of GFP-VIM and RFP-WTαB (*t*-test, the asterisk indicates p=0.02) was also observed. The n inside the bar is the number of cells photobleached.

Representative confocal cell images for FRET-AP are shown in Figure 4 for GFP-VIM and RFP-WTαB and in Figure 5 for GFP-VIM and RFP-R120GαB. The calculated FRET efficiency values were plotted in Figure 6. Significant differences in transfer efficiencies were observed among the various pairs (p=0.0013), and approximately a twofold increase in the transfer efficiency is observed for vimentin and R120G αB-crystallin compared with vimentin and WT αB crystallin (p=0.02).

## Discussion

We have been using sensitized emission to detect FRET [22,23], but recently we found that the acceptor-photobleaching method is more simple. It requires fewer cell samples and involves fewer data manipulations. After preliminary experiments with controls, data acquisition becomes quite straightforward. The choice of donor-acceptor pair is very important; the GFP-RFP we used before gave a satisfactory result. Both GFP and RFP (DsRED) chromophores are sufficiently photostable during imaging. In fact, a complete photobleaching of RFP chromophores is difficult to achieve in a short time. However, a longer bleaching time will complicate imaging results because the samples shift during the time between taking pre- and post-bleach images. For this reason, we used only 45 s of bleaching time to obtain partially bleached cells in our experiments. After photobleaching, green fluorescence intensity is increased when the two target proteins interact because of less FRET. The information from transfer efficiency values is basically the same as net FRET values; they reflect the extent of protein–protein interactions.

The nature of the interaction between vimentin and αB-crystallin is not known, but chaperone binding is thought to be involved. The assembly of filament fibers may need αB-crystallin as a chaperone. However, overexpression of R120G αB-crystallin leads to decreased fibrous vimentin and increased aggregation (Figure 1). This cannot be due to an increase of chaperone binding because of the decreased chaperone-like activity of R120G αB-crystallin [28,29]. The more likely mechanism is increased hydrophobic interaction since the R120G αB-crystallin mutant is partially unfolded and has more exposed hydrophobic surfaces [27]. R120G αB-crystallin is susceptible to aggregation [22], and when coexpressed with vimentin the increased hydrophobic interaction renders them aggregated.

The HeLa cell itself expresses endogenous vimentin, but the amount must be overshadowed by the overexpressed tagged protein, and the interaction between the endogenous vimentin and αB-crystallin should not affect FRET measurements. In the cells, other heat shock proteins such as HSP70 and HSP90 were also found to interact with IFs [30,31], but the nature of their interaction is uncertain; a role to maintain filaments from aggregation was proposed [32].

The lens cell cytoskeleton was also found to associate with membranes. An earlier study indicated that newly synthesized vimentin was associated with lens membranes [33]. The same study also found α-crystallin to be associated with lens membranes. Later, α-crystallin was reported to associate with other cytoskeletal proteins (actin and microtubule) [34-36]. These observations indicate that the three lens components (crystallins, membranes, and cytoskeleton) are interrelated; thus the protein complex, a macromolecular assembly, must be responsible for lens-specific functions. Protein–protein interaction may provide information on not only when two proteins interact but also when such interactions are modified. In age-related cataracts or congenital cataracts, protein modifications or mutations are found. Disruption of protein–protein interactions will profoundly change protein or cell functions. Protein–protein interactions in turn are dictated by specific protein conformations; partial unfolding not only destroys the interaction sites but also exposes buried hydrophobic sites.

Another possible mechanism involves IF structures. Some data suggest that the vimentin IF structure is dynamic; IF undergoes subunit exchange [37-39]. The vimentin filament is composed of smaller protofibrils, each of which in turn consists of two smaller protofilaments. Each protofilament consists of tetramers [12], which are assumed to result from the interaction of two dimers. A monomeric vimentin consists of a central α-helical domain with one non-helical NH_2_- (head) and one COOH- (tail) domain. The two monomers are twisted around each other to form a coiled dimer. The head and tail are involved in the end-end and lateral interactions. The dynamic structure of IFs suggests that IFs reorganize in response to cell cycle-specific or differentiation-specific cues. Thus, the presence of aggregation-prone R120G αB-crystallin may interfere with the IF assembly and disassembly process. The dynamic structure of vimentin may also help the formation of the filament network of tagged GFP-VIM since GFP-VIM can participate in the subunit exchange in the filament network of the endogenous vimentin.

There are two other myopathy-associated αB-crystallin mutants, Q151X and 464delCT [40]. Both mutants caused the formation of cytoplasmic aggregates in skeletal muscles, but did not cause cataract. Apparently, the effects of these two mutants on desmin are the same as the R120G mutant, but the effects on vimentin or other IFs are different from the R120G mutant. The mechanism for the different effects is not known and needs further study.

In conclusion, we have demonstrated that the R120G αB-crystallin mutant promotes vimentin aggregation, and FRET photobleaching shows that the mechanism of aggregation is increased protein–protein interactions between vimentin and R120G αB-crystallin.

## References

[1] Sandilands A, Prescott AR, Carter JM, Hutcheson AM, Quinlan RA, Richards J, FitzGerald PG (1995). Vimentin and CP49/filensin form distinct networks in the lens which are independently modulated during lens fibre cell differentiation.. J Cell Sci.

[2] Clark JI, Matsushima H, David LL, Clark JM (1999). Lens cytoskeleton and transparency: a model.. Eye.

[3] Quinlan RA, Sandilands A, Procter JE, Prescott AR, Hutcheson AM, Dahm R, Gribbon C, Wallace P, Carter JM (1999). The eye lens cytoskeleton.. Eye.

[4] Carter JM, Hutcheson AM, Quinlan RA (1995). In vitro studies on the assembly properties of the lens proteins CP49, CP115: coassembly with alpha-crystallin but not with vimentin.. Exp Eye Res.

[5] Prescott AR, Sandilands A, Hutcheson AM, Carter JM, Quinlan RA (1996). The intermediate filament cytoskeleton of the lens: an ever changing network through development and differentiation. A minireview.. Ophthalmic Res.

[6] Quinlan RA, Carte JM, Sandilands A, Prescott AR (1996). The beaded filament of the eye lens: an unexpected key to intermediate filament structure and function.. Trends Cell Biol.

[7] Muchowski PJ, Valdez MM, Clark JI (1999). AlphaB-crystallin selectively targets intermediate filament proteins during thermal stress.. Invest Ophthalmol Vis Sci.

[8] Nicholl ID, Quinlan RA (1994). Chaperone activity of alpha-crystallins modulates intermediate filament assembly.. EMBO J.

[9] Djabali K, de Nechaud B, Landon F, Portier MM (1997). AlphaB-crystallin interacts with intermediate filaments in response to stress.. J Cell Sci.

[10] Wang X, Osinska H, Klevitsky R, Gerdes AM, Nieman M, Lorenz J, Hewett T, Robbins J (2001). Expression of R120G-alphaB-crystallin causes aberrant desmin and alphaB-crystallin aggregation and cardiomyopathy in mice.. Circ Res.

[11] Perng MD, Wen SF, van den Ijssel P, Prescott AR, Quinlan RA (2004). Desmin aggregate formation by R120G alphaB-crystallin is caused by altered filament interactions and is dependent upon network status in cells.. Mol Biol Cell.

[12] Fuchs E, Weber K (1994). Intermediate filaments: structure, dynamics, function, and disease.. Annu Rev Biochem.

[13] Goebel HH (1995). Desmin-related neuromuscular disorders.. Muscle Nerve.

[14] Vicart P, Caron A, Guicheney P, Li Z, Prevost MC, Faure A, Chateau D, Chapon F, Tome F, Dupret JM, Paulin D, Fardeau M (1998). A missense mutation in the alphaB-crystallin chaperone gene causes a desmin-related myopathy.. Nat Genet.

[15] Fardeau M, Godet-Guillain J, Tome FM, Collin H, Gaudeau S, Boffety C, Vernant P (1978). A new familial muscular disorder demonstrated by the intra-sarcoplasmic accumulation of a granulo-filamentous material which is dense on electron microscopy (author's transl).. Rev Neurol (Paris).

[16] Fardeau M, Vicart P, Caron A, Chateau D, Chevallay M, Collin H, Chapon F, Duboc D, Eymard B, Tome FM, Dupret JM, Paulin D, Guicheney P (2000). Familial myopathy with desmin storage seen as a granulo-filamentar, electron-dense material with mutation of the alphaB-cristallin gene.. Rev Neurol (Paris).

[17] Wang X, Klevitsky R, Huang W, Glasford J, Li F, Robbins J (2003). AlphaB-crystallin modulates protein aggregation of abnormal desmin.. Circ Res.

[18] Goldfarb LG, Vicart P, Goebel HH, Dalakas MC (2004). Desmin myopathy.. Brain.

[19] Perng MD, Muchowski PJ, van Den IP, Wu GJ, Hutcheson AM, Clark JI, Quinlan RA (1999). The cardiomyopathy and lens cataract mutation in alphaB-crystallin alters its protein structure, chaperone activity, and interaction with intermediate filaments in vitro.. J Biol Chem.

[20] Chavez Zobel AT, Loranger A, Marceau N, Theriault JR, Lambert H, Landry J (2003). Distinct chaperone mechanisms can delay the formation of aggresomes by the myopathy-causing R120G alphaB-crystallin mutant.. Hum Mol Genet.

[21] Maisel H, editor. The ocular lens: Structure, function, and pathology. New York: Marcel Dekker; 1985

[22] Liu BF, Anbarasu K, Liang JJ (2007). Confocal fluorescence resonance energy transfer microscopy study of protein-protein interactions of lens crystallins in living cells.. Mol Vis.

[23] Liu BF, Liang JJ (2008). Confocal fluorescence microscopy study of interaction between lens MIP26/AQP0 and crystallins in living cells.. J Cell Biochem.

[24] Steer BA, Merrill AR (1994). The colicin E1 insertion-competent state: detection of structural changes using fluorescence resonance energy transfer.. Biochemistry.

[25] Wu P, Brand L (1994). Resonance energy transfer: methods and applications.. Anal Biochem.

[26] Lakowicz J. Principles of fluorescence spectroscopy. New York: Plenum Press; 1983.

[27] Liang JJ, Liu BF (2006). Fluorescence resonance energy transfer study of subunit exchange in human lens crystallins and congenital cataract crystallin mutants.. Protein Sci.

[28] Bova MP, Yaron O, Huang Q, Ding L, Haley DA, Stewart PL, Horwitz J (1999). Mutation R120G in alphaB-crystallin, which is linked to a desmin-related myopathy, results in an irregular structure and defective chaperone-like function.. Proc Natl Acad Sci USA.

[29] Kumar LV, Ramakrishna T, Rao CM (1999). Structural and functional consequences of the mutation of a conserved arginine residue in alphaA and alphaB crystallins.. J Biol Chem.

[30] Liao J, Lowthert LA, Ghori N, Omary MB (1995). The 70-kDa heat shock proteins associate with glandular intermediate filaments in an ATP-dependent manner.. J Biol Chem.

[31] Fostinis Y, Theodoropoulos PA, Gravanis A, Stournaras C (1992). Heat shock protein HSP90 and its association with the cytoskeleton: a morphological study.. Biochem Cell Biol.

[32] Liang P, MacRae TH (1997). Molecular chaperones and the cytoskeleton.. J Cell Sci.

[33] Ramaekers FC, Dunia I, Dodemont HJ, Benedetti EL, Bloemendal H (1982). Lenticular intermediate-sized filaments: biosynthesis and interaction with plasma membrane.. Proc Natl Acad Sci USA.

[34] Xi JH, Bai F, McGaha R, Andley UP (2006). Alpha-crystallin expression affects microtubule assembly and prevents their aggregation.. FASEB J.

[35] Del Vecchio PJ, MacElroy KS, Rosser MP, Church RL (1984). Association of alpha-crystallin with actin in cultured lens cells.. Curr Eye Res.

[36] Gopalakrishnan S, Takemoto L (1992). Binding of actin to lens alpha crystallins.. Curr Eye Res.

[37] Soellner P, Quinlan RA, Franke WW (1985). Identification of a distinct soluble subunit of an intermediate filament protein: tetrameric vimentin from living cells.. Proc Natl Acad Sci USA.

[38] Ngai J, Coleman TR, Lazarides E (1990). Localization of newly synthesized vimentin subunits reveals a novel mechanism of intermediate filament assembly.. Cell.

[39] Angelides KJ, Smith KE, Takeda M (1989). Assembly and exchange of intermediate filament proteins of neurons: neurofilaments are dynamic structures.. J Cell Biol.

[40] Selcen D, Engel AG (2003). Myofibrillar myopathy caused by novel dominant negative alpha B-crystallin mutations.. Ann Neurol.

